# Effect of Garden Cress Seeds Powder and Its Alcoholic Extract on the Metabolic Activity of CYP2D6 and CYP3A4

**DOI:** 10.1155/2014/634592

**Published:** 2014-03-10

**Authors:** Fahad I. Al-Jenoobi, Areej A. Al-Thukair, Mohd Aftab Alam, Fawkeya A. Abbas, Abdullah M. Al-Mohizea, Khalid M. Alkharfy, Saleh A. Al-Suwayeh

**Affiliations:** ^1^Department of Pharmaceutics, College of Pharmacy, King Saud University, P.O. Box 2457, Riyadh 11451, Saudi Arabia; ^2^Department of Pharmacognosy, College of Pharmacy, King Saud University, P.O. Box 2457, Riyadh 11451, Saudi Arabia; ^3^Department of Clinical Pharmacy, College of Pharmacy, King Saud University, P.O. Box 2457, Riyadh 11451, Saudi Arabia

## Abstract

The powder and alcoholic extract of dried seeds of garden cress were investigated for their effect on metabolic activity of CYP2D6 and CYP3A4 enzymes. In vitro and clinical studies were conducted on human liver microsomes and healthy human subjects, respectively. Dextromethorphan was used as a common marker for measuring metabolic activity of CYP2D6 and CYP3A4 enzymes. In in vitro studies, microsomes were incubated with NADPH in presence and absence of different concentrations of seeds extract. Clinical investigations were performed in two phases. In phase I, six healthy female volunteers were administered a single dose of dextromethorphan and in phase II volunteers were treated with seeds powder for seven days and dextromethorphan was administered with last dose. The O-demethylated and N-demethylated metabolites of dextromethorphan were measured as dextrorphan (DOR) and 3-methoxymorphinan (3-MM), respectively. Observations suggested that garden cress inhibits the formation of DOR and 3-MM metabolites. This inhibition of metabolite level was attributed to the inhibition of CYP2D6 and CYP3A4 activity. Garden cress decreases the level of DOR and 3-MM in urine and significantly increases the urinary metabolic ratio of DEX/DOR and DEX/3-MM. The findings suggested that garden cress seeds powder and ethanolic extract have the potential to interact with CYP2D6 and CYP3A4 substrates.

## 1. Introduction

The consumption of herbs is common either in the form of food or as traditional medicine. Since decades, the use of herbs was considered safe. However, in recent years, there are large numbers of reports related to herbal toxicity and herb's effects on basic activities such as modulation of drug metabolizing enzymes and drug transporters and interference with bioavailability and pharmacokinetics of concomitantly administered therapeutic substances [1–3]. The metabolic enzyme modulation also leads to incidences of altered bioavailability and pharmacokinetics of therapeutic substrates. The consequences will be more serious with substrates having narrow therapeutic index [4–6]. In human, most of the drugs are metabolized by CYP3A4, CYP2D6, CYP2C9/10, CYP2C19, CYP2E1, and CYPlA2 [7–9]. Han et al. reported that Rhizoma Coptidis showed in vitro inhibition of CYP2D6 [10]. Corynoline, an isoquinoline alkaloid, showed strong inhibitory effects on the activities of CYP3A4 and CYP2C9 [11]. Myricetin inhibited the liver metabolizing enzymes CYP3A4 and CYP2C9 [12]. St. John's wort induces CYP3A4 and P-glycoprotein and reduces the blood level of cyclosporine, tacrolimus, digoxin, amitriptyline, midazolam, warfarin, indinavir, phenprocoumon, and theophylline [6, 13, 14].

Garden cress (*Lepidium sativum *L.) belongs to the family Cruciferae [15]. The seeds comprise 33–54% of carbohydrates, 25% of protein, 14–24% of lipids, and 8% of crude fiber. The major abundant amino acids of seeds were aspartic and glutamic acids. Potassium was the most abundant mineral [16]. Garden cress seed also contains 20–25% yellowish semidrying oil. The major fatty acid of oil is alpha-linolenic acid (34.0%). The oil also comprises polyunsaturated fatty acids (46.8%) and monounsaturated fatty acids (37.6%) and antioxidants, such as tocopherols and carotenoids [17, 18]. Seven imidazole alkaloids; lepidine B, C, D, E, and F (dimeric); and two new monomeric alkaloids semilepidinoside A and B were reported in seeds of* L. sativum *[18, 19]. The herb is used for cough, vitamin C deficiency, constipation, and poor immunity and as a diuretic. The modern practitioners of Indian medicine consider the seeds useful in dysenteric diarrhea as well as in febrile and catarrhal infections. The seeds are also considered to be lactagogue [18, 20].

The objective of present investigation was to evaluate the metabolizing enzyme modulating potential of* Lepidium sativum.* The metabolic activities of enzymes CYP3A4 and CYP2D6 were assessed. The human liver microsomes were employed for in vitro investigation, while in vivo study was conducted on healthy human subjects. Dextromethorphan (DEX) was used as common marker for both enzymes. The DEX and its metabolites were estimated in the urine and urinary metabolic ratios of dextromethorphan and its metabolites were calculated. DEX is extensively metabolized in the liver by N-demethylation and O-demethylation. The O-demethylation of DEX is primarily catalyzed by CYP2D6 and forms dextrorphan (DOR) [21]. The N-demethylation of DEX is mediated by CYP3A4 to form 3-methoxymorphinan (3-MM) [22].

## 2. Materials and Methods

Dextromethorphan hydrobromide, dextrorphan hydrobromide, and 3-methoxymorphinan hydrobromide were purchased from ICN Biomedicals Inc., Warrenale, USA. Dextromethorphan hydrobromide syrup was obtained from Riyadh Pharma Medical and Cosmetic Products Co. Ltd., Riyadh, Saudi Arabia. Human liver microsomes (protein concentration of 20 mg/mL) were purchased from Human Biologics International LLC (HBI, Scpttsdale, USA), shipped in small vials on dry ice, and stored at −80°C. Nicotinamide adenine dinucleotide phosphate (NADPH) and *β*-glucuronidase (76,800 U/mL) were purchased from Helix Pomatia, ICN Biomedicals Inc., Costa Mesa, AC, USA. Garden cress seeds were purchased from local Saudi Market. Codeine and betaxolol were of USP reference standard. General purpose reagents (GPR) were used for extraction processes, while HPLC grade solvents were used for HPLC determinations. All other materials are of analytical grade.

### 2.1. Extract of Plant Material

Dried garden cress seeds were finely powered and exhaustively macerated. The cold maceration was carried out for five days with ethanol. Extract was filtered and concentrated under reduced pressure at 40°C by using rotatory evaporator. Weighed concentrated extract was serially diluted with ethanol (96%) to give stock solutions of concentrations of 1.25, 2.5, 5, 25, and 50 mg/mL. These diluted ethanolic stock solutions were stored in refrigerator.

### 2.2. Microsomal Incubation

Dextromethorphan (DEX) dissolved in methanol was transferred into dried and clean Eppendorf tubes at final reaction concentration of 25 *μ*M. The methanol was evaporated by using nitrogen. Human liver microsomes (0.25 mg/mL protein conc.) and appropriate volume of potassium phosphate buffer (0.1 M, pH 7.4) were transferred into DEX loaded tubes. The loaded composition (DEX, microsomes, and buffer) was gently mixed and preincubated in a shaker water bath at 37°C for 3 minutes. The in vitro metabolic reaction was initiated in a final volume of 0.5 mL by adding 1 mM NADPH, in absence and presence of garden cress seeds extract (at concentrations of 10, 25, 50, and 100 *μ*g/mL). In vitro metabolic reaction was run for about 30 minutes. Reaction was terminated by adding 70% perchloric acid (10 *μ*L) with vigorous shaking for 2-3 minutes. The experiments were repeated in triplicate at each concentration of extract. Codeine (25 *μ*L) was added as an internal standard to each tube. The mixture was centrifuged at 10000 rpm for about 15 minutes. Supernatant was separated and transferred to a clean vial and injected for HPLC analysis of metabolites.

### 2.3. Clinical Study

Nonsmoking, healthy female volunteers (*n* = 6) of 18–35 years of age were selected to participate in this study. Details of clinical study protocol were explained to the volunteers and written informed consent was received from each one of them. Study protocol was approved by Ethics Committee at College of Medicine, King Saud University, Riyadh. Subjects were asked to refrain from caffeine and caffeine containing products, for at least 24 hours before study. Furthermore, the volunteers were also asked not to take any other traditional/conventional/herbal medications or grapefruit/grapefruit comprising foodstuff for at least two weeks before and during the study. The study was conducted in two phases with two weeks washout period. In phase I, all subjects received a single oral dose of DEX (30 mg) by administering 10 mL of 15 mg/5 mL DEX HBr syrup. The subjects were not allowed to eat for two hours before and after dosing. Subjects were instructed to empty their bladder before dosing. The urine samples were collected for eight hours after DEX administration, and urine aliquots were stored at −20°C until analyzed. In phase II, the powder of garden cress seed was administered in a dose of 7.5 gm twice daily for seven consecutive days. On the last dosing day, subjects received DEX (10 mL of 15 mg/5 mL syrup) and powder of garden cress seeds, concurrently. The subjects were not allowed to eat for two hours before and after dosing. Subjects were instructed to empty their bladder before dosing. The urine samples were collected and stored at −20°C until analyzed. The concentrations of DEX and its metabolites were estimated by using HPLC methods. The urinary metabolic ratios of DEX/3-MM and DEX/DOR were used as indices to metabolic activities of CYP3A4 and CYP2D6 enzymes, respectively.

### 2.4. Preparation of Urine Samples

The human urine samples were hydrolyzed by using *β*-glucuronidase (19200 U/mL, incubated for 18 hours) to obtain unconjugated form of DEX and its metabolites. Hydrolyzed urine sample (1 mL) was vigorously mixed with 5 mL organic extracting solvent system (diethyl ether/chloroform/propranolol, 20 : 9 : 1 v/v/v). This vigorously mixed mixture was centrifuged at 14500 g for 10 minutes. The organic layer was separated and again vigorously mixed with 300 *μ*L of 0.1 N hydrochloric acid and centrifuged at 14500 g for 10 minutes. The aqueous layer was separated and transferred into HPLC vials for analysis.

### 2.5. Determination of DEX and Its Metabolites

The analysis of DEX metabolites was performed on Shimadzu Class-VPV 5.02 instrument. Two distinct HPLC methods were adopted for analysis of DEX and its metabolites in human liver microsomes and the urine of human volunteers [23, 24]. In vitro microsomal analytes were eluted on a Nova-Pak phenyl column (5 *μ*m, 150 × 3.9 mm) by using a mobile phase composed of a 75 : 25 mixture of acetonitrile and water (1.5% glacial acetic acid and 0.1% triethylamine), flowing at 1 mL/min. The urine samples were analyzed by using a Zorbax SB-CN column (5 *μ*m, 250 × 4.6 mm). The mobile phase for urine samples was a mixture of water (1.5% glacial acetic acid and 0.1% triethylamine) and acetonitrile (87.5 : 12.5 v/v). The pH of the mobile phase was adjusted to 3 using orthophosphoric acid. Analytes were monitored using a fluorescence detector at excitation and emission wavelengths of 280 and 330 nm, respectively. The calibration curves for microsomal metabolite and dextromethorphan were constructed.

### 2.6. Statistical Analysis

For in vitro microsomal study, formation of metabolites from DEX in presence of garden cress extract was compared to that of control by using one-way analysis of variance (ANOVA) and a post hoc Scheffe's multiple comparison test with a significant *P* value ≤0.05. Statistical analysis of results obtained from clinical study was performed by using paired Student's *t*-test. Differences were considered statistically significant when *P* values were ≤ 0.05. Statistical analysis was conducted using Graph-Pad Prism version 3.0 for Windows (San Diego, CA, USA).

## 3. Results

**Figure 1:**
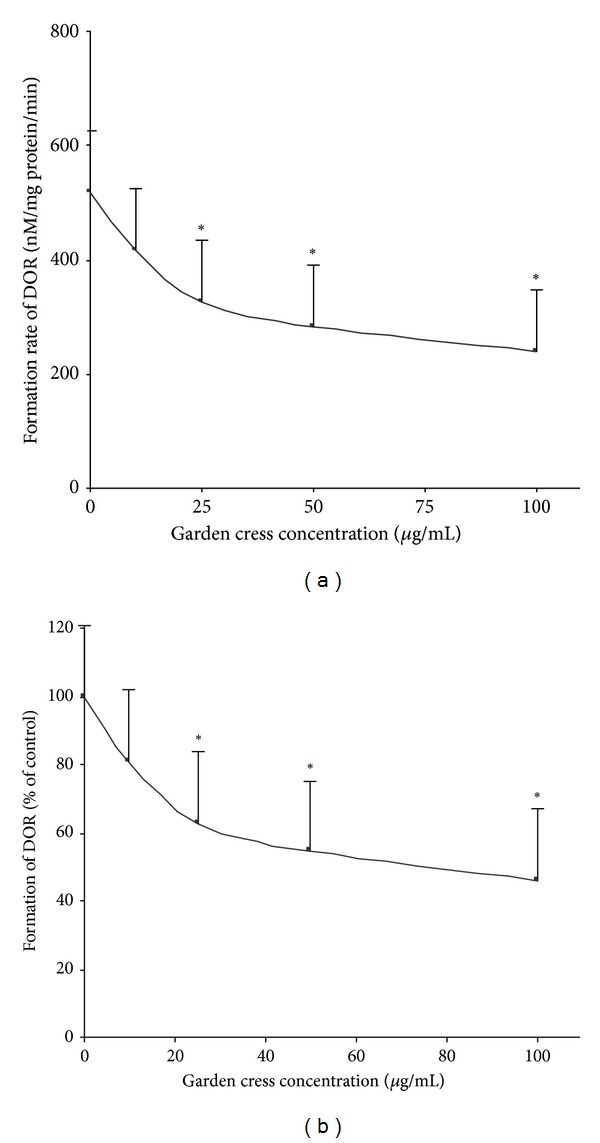
Effect of garden cress on the formation of DOR from DEX in human liver microsomes (*n* = 3, mean ± SD). Formation of the metabolite is expressed as (nM/mg protein/min) in (a) and percent of control in (b). *P ≤ 0.05.

**Figure 2:**
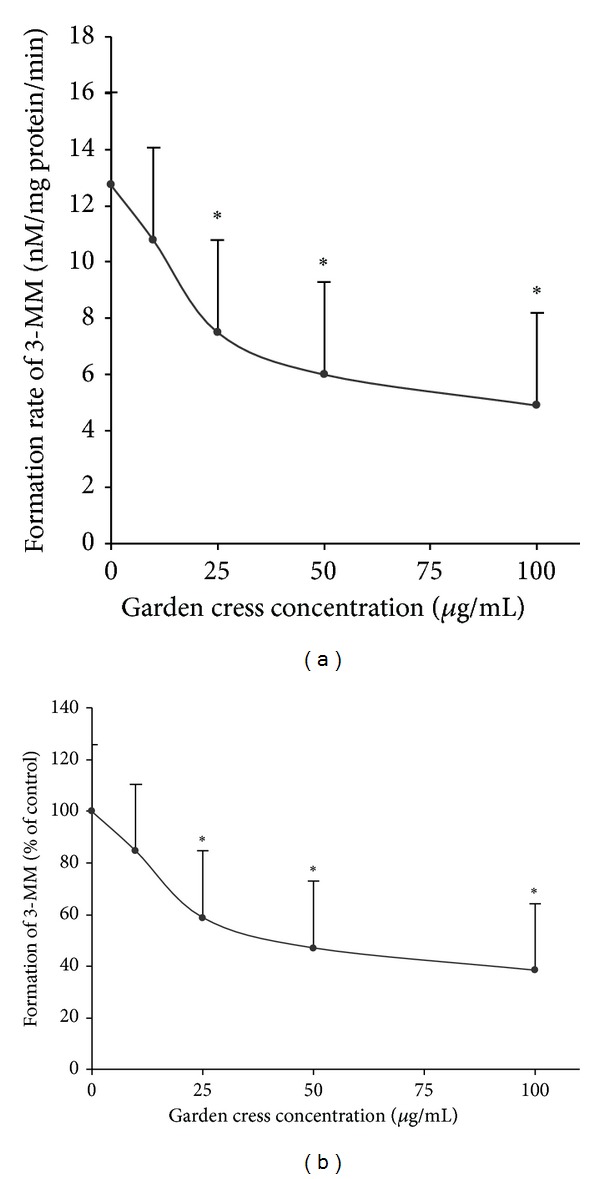
Effect of garden cress on the formation of 3-MM from DEX in human liver microsomes (*n* = 3, mean ± SD). Formation of the metabolite is expressed as (nM/mg protein/min) in (a) and percent of control in (b). *P ≤ 0.05.

The effect of garden cress seeds powder and its ethanolic extract was investigated on CYP2D6 and CYP3A4 mediated metabolism of dextromethorphan. The level of DEX metabolites dextrorphan (DOR) and 3-methoxymorphinan (3-MM) was determined in the absence and presence of garden cress. The investigations were carried out in vitro by using human liver microsomes and healthy volunteers were recruited for in vivo study. Ethanolic extract of garden cress seeds showed concentration-dependent inhibition of DOR and 3-MM formation. In vitro results are illustrated in Figures 1 and 2.  Figure 1(a) represents the formation of DOR (nM/mg protein/min).  Figure 2(a) represents the formation of 3-MM (mM/mg protein/min). At lower concentration (10 *μ*g/mL), ethanolic extract produced insignificant effect, and about 20% inhibition of DOR and 3-MM level was observed. Ethanolic extract at concentrations of 25 and 50 *μ*g/mL produced statistically significant inhibition of about 37–45% DOR and about 42–52% 3-MM levels, respectively. The maximum inhibition (about 50%) of DOR formation was observed at highest tested concentration of garden cress extract (100 *μ*g/mL). Ethanolic extract also produced a statistically significant inhibition of 3-MM level (by 60%) at 100 *μ*g/mL.

Clinical observations showed that garden cress seeds powder produced a remarkable inhibitory effect on the DOR level in urine. The quantitative reduction in the amount of DOR excreted in urine in presence of herb was about 30%. The urine metabolic ration of DEX/DOR was increased significantly in presence of garden cress seed powder (see Table 1). Table 1 summarizes the ratio of DEX and its metabolites excreted in urine before and after administration of garden cress seed powder. As presented in Table 1, the metabolic ratio of DEX/3-MM was also increased significantly in presence of garden cress seeds powder. The inhibitory effect of garden cress on 3-MM level in urine was statistically significant; about 40% inhibition was recorded.

## 4. Discussion

The in vitro and in vivo metabolic activities of enzymes CYP2D6 and CYP3A4 were assessed in presence and absence of garden cress seed powder and ethanolic extract. These investigations were carried out to demonstrate the enzymes modulating potential of garden cress seeds powder and ethanolic extract. Dextromethorphan was used as a common probe for CYP2D6 and CYP3A4 enzymes [25–28]. The O-demethylation of dextromethorphan was used to assess the in vitro and in vivo metabolic activity of CYP2D6. The N-demethylation of dextromethorphan was used to assess the activity of CYP3A4. Garden cress ethanolic extract significantly inhibited the activity of hepatic microsomal enzymes CYP2D6 and CYP3A4, which was represented by decreased level of metabolites DOR and 3-MM in in vitro experiments. The effect was observed as concentration dependent. The highest decrease in the metabolite levels was observed at the highest tested concentration of extract. These findings suggested that garden cress have potential to inhibit metabolic activity of CYP2D6 and 3A4. A clinical study was designed to evaluate the effect of garden cress on metabolic activity of CYP2D6 and 3A4. Therefore, the effect was evaluated in two phases on healthy volunteers. The garden cress significantly diminishes the level of DOR and 3-MM metabolites excreted in urine. Furthermore, the urinary metabolic ratio of DEX/DOR and DEX/3-MM was also enhanced. The high intraindividual variability of DEX/3-MM urinary metabolic ratio was observed, which may be because of partial N-demethylation of DEX by CYP3A4. These observations indicated that garden cress has remarkable inhibitory effect on the activities of human CYP2D6 and CYP3A4 enzymes. The results of clinical study were generally consistent with the results obtained from in vitro microsomal investigations.

## 5. Conclusion

Caution should be warranted when garden cress seeds are consecutively administered with therapeutics medicines metabolized by CYP2D6 and CYP3A4 enzymes. Special attention is required if the substrate is of narrow therapeutic index such as carbamazepine and cyclosporine. Further investigations are suggested to isolate the active constituents responsible for this inhibitory effect and to find exact mechanism of interaction between garden cress and CYP2D6 and 3A4 substrates.

## Figures and Tables

**Table 1:** Urinary metabolic ratio (MR) of DEX with its metabolites (*n* = 6).

Subjects	Urinary metabolic ratio DEX/DOR		Urinary metabolic ratio DEX/3-MM	
	MR, control	MR, phase-II	MR, control	MR, phase-II
1	0.034	0.049	2.387	3.388
2	0.067	0.086	4.382	8.0586
3	0.030	0.060	1.382	2.781
4	0.028	0.038	0.379	0.519
5	0.032	0.044	2.118	3.666
6	0.073	0.090	3.770	5.160
Mean	0.044	0.061	2.403	3.929
SD	0.020	0.022	1.483	2.524
*P*	0.002		0.025	

## References

[1] Efferth T, Kaina B (2011). Toxicities by herbal medicines with emphasis to traditional Chinese medicine. *Current Drug Metabolism*.

[2] Chien C-F, Wu Y-T, Lee W-C, Lin L-C, Tsai T-H (2010). Herb-drug interaction of *Andrographis paniculata* extract and andrographolide on the pharmacokinetics of theophylline in rats. *Chemico-Biological Interactions*.

[3] Yang X-X, Hu Z-P, Duan W, Zhu Y-Z, Zhou S-F (2006). Drug-herb interactions: eliminating toxicity with hard drug design. *Current Pharmaceutical Design*.

[4] Müller AC, Kanfer I (2011). Potential pharmacokinetic interactions between antiretrovirals and medicinal plants used as complementary and African traditional medicines. *Biopharmaceutics and Drug Disposition*.

[5] Izzo AA, Ernst E (2001). Interactions between herbal medicines and prescribed drugs: a systematic review. *Drugs*.

[6] Barone GW, Gurley BJ, Ketel BL, Abul-Ezz SR (2001). Herbal supplements: a potential for drug interactions in transplant recipients. *Transplantation*.

[7] Zhou S-F (2008). Drugs behave as substrates, inhibitors and inducers of human cytochrome P450 3A4. *Current Drug Metabolism*.

[8] Lee SY, Lee JY, Kang W (2013). Cytochrome P450-mediated herb-drug interaction potential of Galgeun-tang. *Food and Chemical Toxicology*.

[9] Lee SY, Lee JY, Kang W (2013). *In vitro* and *in vivo* assessment of cytochrome P450-mediated herb-drug interaction of Ssang-hwa-tang. *Food Chemistry*.

[10] Han Y-L, Yu H-L, Li D (2011). *In vitro* inhibition of huanglian [*Rhizoma coptidis* (L.)] and its six active alkaloids on six cytochrome P450 isoforms in human liver microsomes. *Phytotherapy Research*.

[11] Fang Z-Z, Zhang Y-Y, Ge G-B (2011). Identification of cytochrome P450 (CYP) isoforms involved in the metabolism of corynoline, and assessment of its herb-drug interactions. *Phytotherapy Research*.

[12] Choi D-H, Li C, Choi J-S (2010). Effects of myricetin, an antioxidant, on the pharmacokinetics of losartan and its active metabolite, EXP-3174, in rats: possible role of cytochrome P450 3A4, cytochrome P450 2C9 and P-glycoprotein inhibition by myricetin. *Journal of Pharmacy and Pharmacology*.

[13] Hu Z, Yang X, Ho PCL (2005). Herb-drug interactions: a literature review. *Drugs*.

[14] Izzo AA (2005). Herb-drug interactions: an overview of the clinical evidence. *Fundamental and Clinical Pharmacology*.

[15] Raval ND, Pandya TN (2011). Pharmacognostic study of *Lepidium sativum* Linn (*Chandrashura*). *Ayu*.

[16] Gokavi SS, Malleshi NG, Guo M (2004). Chemical composition of garden cress (*Lepidium sativum*) seeds and its fractions and use of bran as a functional ingredient. *Plant Foods for Human Nutrition*.

[17] Diwakar BT, Dutta PK, Lokesh BR, Naidu KA (2010). Physicochemical properties of garden cress (*lepidium sativum* l.) seed oil. *Journal of the American Oil Chemists’ Society*.

[18] Datta PK, Diwakar BT, Viswanatha S, Murthy KN, Naidu KA (2011). Safety evaluation studies on Garden cress (*Lepidium sativum* L.) seeds in Wistar rats. *International Journal of Applied Research in Natural Products*.

[19] Maier UH, Gundlach H, Zenk MH (1998). Seven imidazole alkaloids from *Lepidium sativum*. *Phytochemistry*.

[20] Rehman N-U, Khan A-U, Alkharfy KM, Gilani A-H (2012). Pharmacological basis for the medicinal use of *Lepidium sativum* in airways disorders. *Evidence-based Complementary and Alternative Medicine*.

[21] Barnhart JW (1980). The urinary excretion of dextromethorphan and three metabolites in dogs and humans. *Toxicology and Applied Pharmacology*.

[22] Gorski JC, Jones DR, Wrighton SA, Hall SD (1994). Characterization of dextromethorphan N-demethylation by human liver microsomes: contribution of the cytochrome P450 3A (CYP3A) subfamily. *Biochemical Pharmacology*.

[23] Bendriss E-K, Markoglou N, Wainer IW (2001). High-performance liquid chromatography assay for simultaneous determination of dextromethorphan and its main metabolites in urine and in microsomal preparations. *Journal of Chromatography B*.

[24] Min DI, Ku Y-M, Vichiendilokkul A, Fleckenstein LL (1999). A urine metabolic ratio of dextromethorphan and 3-methoxymorphinan as a probe for CYP3A activity and prediction of cyclosporine clearance in healthy volunteers. *Pharmacotherapy*.

[25] Yu A, Haining RL (2001). Comparative contribution to dextromethorphan metabolism by cytochrome P450 isoforms *in vitro*: can dextromethorphan be used as a dual probe for both CYP2D6 and CYP3A activities?. *Drug Metabolism and Disposition*.

[26] Ducharme J, Abdullah S, Wainer IW (1996). Dextromethorphan as an *in vivo* probe for the simultaneous determination of CYP2D6 and CYP3A activity. *Journal of Chromatography B*.

[27] Funck-Brentano C, Boëlle P-Y, Verstuyft C, Bornert C, Becquemont L, Poirier J-M (2005). Measurement of CYP2D6 and CYP3A4 activity *in vivo* with dextromethorphan: sources of variability and predictors of adverse effects in 419 healthy subjects. *European Journal of Clinical Pharmacology*.

[28] Jones DR, Gorski JC, Hamman MA, Hall SD (1996). Quantification of dextromethorphan and metabolites: a dual phenotypic marker for cytochrome P450 3A4/5 and 2D6 activity. *Journal of Chromatography B*.

